# Boosting the Valorization of Pigmented Corn Cobs Through Solid-State Fermentation with *Saccharomyces cerevisiae*

**DOI:** 10.3390/molecules31111895

**Published:** 2026-06-01

**Authors:** María Cristina Agustín-Chávez, Ulises Ramírez-Esparza, Emilio Ochoa-Reyes, Juan A. Ascacio-Valdés, Esteban Sánchez-Chávez, Cristóbal N. Aguilar, Lilia Arely Prado-Barragán, José Juan Buenrostro-Figueroa

**Affiliations:** 1 Biotechnology and Bioengineering Laboratory, Research Center in Food and Development, Delicias 33089, Chihuahua, Mexico; cristina.agustin0201@gmail.com (M.C.A.-C.); uramirez223@estudiantes.ciad.mx (U.R.-E.); emilio.ochoa@ciad.mx (E.O.-R.); 2 Bioprocesses & Bioproducts Research Group, Food Research Department, School of Chemistry, Universidad Autónoma de Coahuila, Saltillo 25280, Coahuila, Mexico; cristobal.aguilar@uadec.edu.mx; 3 Plant Physiology Laboratory, Research Center in Food and Development, Delicias 33089, Chihuahua, Mexico; esteban@ciad.mx; 4 Solid Fermentation Pilot Plant, Biotechnology Department, Autonomous Metropolitan University—Iztapalapa, Mexico City 09340, Mexico; lapb@xanum.uam.mx

**Keywords:** agro-industrial by-products, enzymes production, sustainable bioprocessing, phenolic profile, biotransformation

## Abstract

Pigmented corn cobs (PCC) are a rich source of bioactive phenolic compounds (BPC) that can be obtained through sustainable solid-state fermentation (SSF). In this work, PCC were evaluated as a support for SSF using *Saccharomyces cerevisiae*, focusing on the effects of culture time and aeration on BPC recovery. Additionally, the relationships among BPC content, antioxidant capacity (AC), and the production of selected industrial enzymes were evaluated. The physicochemical properties of PCC proved suitable for SSF. After 12 h of fermentation, condensed phenols increased by 120.37% and showed positive correlations with AC, β-glucosidase and tannase activities. A total of 33 compounds, including anthocyanins, flavonols, and hydroxycinnamic acids, were identified via HPLC-MS during SSF. Notable changes in the phenolic profile were observed as a result of enzymatic biodegradation and biotransformation mediated by *S. cerevisiae*. Compounds such as caffeic acid and *p*-coumaric acid were highlighted due to their biological activity and industrial relevance. Forced aeration played a key role in SSF performance by enhancing enzyme production and BPC release. The incorporation of aeration boosts enzyme production and BPC release, thereby improving process efficiency. Overall, SSF represents a sustainable strategy for PCC valorization within a circular economy framework.

## 1. Introduction

Currently, corn is grown in various colors, including blue, black, cherry, purple, and red. The degree of pigmentation is due to the presence of bioactive phenolic compounds (BPC), such as anthocyanins, which are found in higher concentrations in corn cobs [1]. Corn consists of the stalk, seed or corn kernel, cob, leaves, ear, and stigmas, also known as corn silk [2]. Pigmented corn cobs (PCC), an agricultural by-product derived from corn, represent 18% of the total weight of the crop and are an important source of essential nutrients such as proteins and minerals, as well as dietary fiber and BPC with various biological properties, such as antioxidants [3,4].

The BPC are food constituents that exert beneficial effects on human health. However, these compounds can be trapped in plant cell walls, making them difficult to release and utilize [5]. Conventional extraction methods, typically based on solvents and high temperatures, are often inefficient, may damage BPC, generate effluents, and yield low recovery of bound compounds [6]. As an alternative to these, solid-state fermentation (SSF) has emerged as an environmentally friendly technique that involves producing microbial enzymes that aid in the degradation of cell wall components, thereby promoting the recovery of BPC [7,8].

The SSF allows agro-industrial waste to be used as a substrate/support, offering sustainable alternatives for the recovery of industrially valuable biocompounds, such as BPC and enzymes. In SSF, the selection of appropriate microorganisms is critical. Although fungi are the most commonly employed microorganisms due to their adaptability and capacity to produce extracellular enzymes, the potential application of yeasts such as *Saccharomyces cerevisiae* has recently gained attention. This microorganism can produce enzymes and promote the release of BPC from different substrates, including pomegranate and rambutan peels [9,10]; however, its application in PCC has not yet been investigated.

Despite the recognized phenolic potential of PCC, limited information is available regarding the use of SSF to enhance the enzymatic release of bound phenolics from this substrate. Therefore, the aim of this study was to evaluate the effect of SSF with *S. cerevisiae* on BPC release, antioxidant capacity (AC), and enzymatic activity during the biotransformation of PCC.

## 2. Materials and Methods

### 2.1. Preparation of the Substrate

Pigmented corn cobs (PCC), a by-product of blue corn (*Zea mays* L.) obtained from an experimental cultivation harvested during the spring-summer 2023 season, were provided by the Universidad Autónoma Agraria Antonio Narro. The material was collected in black bags and transported to the Biotechnology and Bioengineering Laboratory at CIAD Delicias, where it was dehydrated (50 °C, 48–72 h) and ground to a particle size of 1–2 mm. The processed material was stored in light-protected containers at room temperature until use.

### 2.2. Physicochemical Characterization of PCC

Proximate composition analysis, including carbohydrate, protein, fat, fiber, ash, and moisture content, was performed according to the procedures established by the Association of Official Analytical Chemists (AOAC) [11]. The water absorption capacity (WAC), critical humidity point (CHP), and maximum moisture content (MMC) were determined according to Ordoñez-Cano et al. [12]. For WAC determination, 1.5 g of PCC was placed in a 50 mL tube containing 15 mL of distilled water. The sample was vortex-mixed for 1 min and centrifuged (3000× *g*, 25 °C, 10 min). After centrifugation, the supernatant was discarded, and the hydrated sample was collected and weighed. Results were expressed as grams of gel per gram of dry mass (g gel/g dm). For the CHP assay, 1 g of the gel obtained from the WAC test was dehydrated using a thermobalance (MB120 Ohaus^®^, Parsippany, NJ, USA) at 105 °C, while changes in weight and moisture content were continuously recorded. Drying curves were subsequently generated. The MMC was calculated using the PCC solids balance, moisture content, and WAC values according to Equations (1)–(5).

General balance

(1) M1+M2=M3

Solid and water balance

(2) $$x_{a\;}M_{1}=\left(\frac{M_{1}}{100}*Moisture\;content\;\%\right)$$

(3) $$1-x_{a}M_{1}=x_{s}M_{1}$$

(4) $$M_{1}*\;x_{S}M_{1}=M_{3}*x_{s\;}M_{3}$$

(5) $$1-x_{s}M_{3\;}=x_{a}M_{3}$$

where *M*_1_ = dry sample weight (g) of WAC: *M*_2_ = water (g): *M*_3_ = gel weight (g) of WAC: *x*_a_*M*_1_ = water fraction in *M*_1_: *x*_a_*M*_3_ = water fraction in *M*_3_: *x*_s_*M*_1_ = solids fraction in *M*_1_: *x*_s_*M*_3_ = solids fraction in *M*_3_.

### 2.3. Microorganism and Inoculum Preparation

The yeast *S. cerevisiae* 227 (Collection of Instituto Tecnológico de Durango, Mexico) was used. This yeast was reactivated on potato-dextrose agar (PDA_Bioxon^®^, Mexico City, Mexico) plates and incubated at 30 °C for 5 days (Heratherm IMC 18, Thermo Scientific^®^, Langensebold, Hesse, Germany). Then, propagation was performed in 250 mL Erlenmeyer flasks with 30 mL of PDA, and incubated for 5 days at 30 °C. The new cells were then collected with a sterile 0.01% (*v*/*v*) Tween-80 solution and counted in a Neubauer chamber.

### 2.4. Solid-State Fermentation

**Figure 1:**
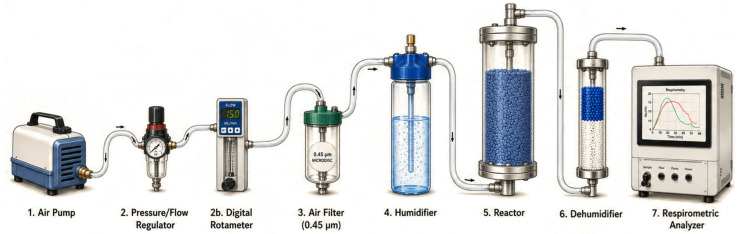
Schematic representation of the forced-aeration SSF system used in this study, including the air supply (1–2), filtration (3), humidification (4), bioreactor (5), dehumidification (6), and respirometric monitoring unit (7).

Six grams of PCC were weighed and impregnated with 9 mL of culture medium (10 g/L peptone, 10 g/L yeast extract, and 0.230 g/L NaCl; pH 6.0), previously inoculated with *S. cerevisiae* (1 × 10^6^ cells g^−1^), to achieve a moisture content of 60%. The resulting wet mixture was packed into polypropylene column reactors (3.5 × 10 cm) and incubated at 30 °C for 72 h under two forced aeration conditions (0 and 1 L/Kg_wm_ min (liters of air per kilogram of wet mass per minute)). For the forced aeration system (Figure 1), sterile saturated air was continuously supplied after passing through a 0.45 µm Whatman filter, and airflow was calibrated using an electronic flowmeter (Agilent Technologies 5067-0223, Santa Clara, CA, USA). At 12 h intervals, three experimental units were sampled to determine the moisture content by drying at 105 °C until a constant weight was achieved, and extracts were collected for subsequent analyses. The 0 h treatment corresponded to the unfermented PCC substrate and served as the initial control condition prior to the SSF process. Microbial growth in the forced aerated system was simultaneously monitored through CO_2_ production and O_2_ consumption. Samples were stored at −18 °C until analysis. All analyses were performed in triplicate.

### 2.5. Extraction of Samples

#### 2.5.1. Phenolic Extracts

The extracts were obtained following the method by Fernandez-Aulis et al. [13]. Briefly, 0.5 g of the sample was mixed with 5 mL of methanol, distilled water, and lactic acid solution (80:19:1 *v*/*v*/*v*). After homogenization, the mixture was sonicated for 5 min using an ultrasonic cleaner (VWR International, 150D, New York, NY, USA), filtered through Whatman^®^ filter paper (25 µm) (Cytiva, Marlborough, MA, USA), and centrifuged at 5700 rpm for 5 min (5805F, Hamburg, Germany). The supernatant was collected and stored in 2 mL vials at −20 °C until analysis.

#### 2.5.2. Enzymatic Extracts

For extract preparation, 3 g of sample (PCC) were mixed with 7 mL of citrate buffer (0.05 M, pH 5.0). The mixture was filtered through Whatman^®^ filter paper (25 µm) and centrifuged (5700 rpm, 5 min). The resulting extracts were stored in 2 mL vials at −20 °C until analysis.

### 2.6. Analytical Methods

#### 2.6.1. Respirometry Analysis

Microbial growth was indirectly estimated using kinetic parameters according to the method described by Méndez-González et al. [14]. The exhaust gas from the bioreactor was dried using silica gel and subsequently analyzed using a respirometric analyzer. The CO_2_ production rate (CPR) was calculated from the concentration gradients between the inlet and outlet air streams of the bioreactor and expressed as mg/g of initial dry matter (gidm). Total CO_2_ production (TCP) was determined by integrating the CO_2_ production rate over time. The lag phase (Lag) was estimated as the x-intercept from the linear regression of Ln(CO_2_ production) versus time. Kinetic parameters, including the specific CO_2_ production rate (μ), initial CO_2_ production (CO_2o_), and total CO_2_ production (CO_2max_), were determined using the logistic model (Equations (6) and (7)) through the generalized reduced gradient method and linear regression analysis.

(6) $${CO}_{2}=\frac{{CO}_{2max}}{1+\left[\left(\frac{{CO}_{2max}}{{CO}_{2o}}\right)-1\right]e^{-\mu t}}$$

(7) $$f\left(t\right)=\frac{{CO}_{2max}}{1+C^{-\mu t}}$$

#### 2.6.2. Hydrolysable Phenols (HP)

A sample (20 µL) was mixed with 20 µL of 2N Folin–Ciocalteu reagent and allowed to react for 5 min. Subsequently, 20 µL of 0.01 M sodium carbonate was added to stop the reaction, and the mixture was kept in the dark for another 5 min. The solution was then diluted with 125 µL of distilled water, and the absorbance at 790 nm was measured using a microplate reader (Multiskan GO, Thermo Scientific, Vantaa, FI) [15]. Results were expressed as mg of gallic acid equivalents (GAE) per gram of dry matter (g dm^−1^), based on a gallic acid curve from 0 to 200 mg L^−1^.

#### 2.6.3. Condensed Phenols (CP)

Condensed phenols were determined according to Ordoñez-Cano et al. [12]. For that, 250 µL of extract was mixed with 1.5 mL of HCl-Butanol (1:9 *v*/*v*) and 50 µL of ferric reagent (2 mL of HCl and 0.2 g of ferric ammonium sulfate adjusted to 10 mL with distilled water). The mixture was sealed and boiled for 40 min, then allowed to cool to room temperature (25 °C). Subsequently, 200 µL of the reaction mixture was transferred to a microplate well, and the absorbance was recorded at 460 nm. The results were expressed as mg of catechin equivalents (CE) per gram of dry matter (g dm^−1^), using a catechin curve from 0 to 1000 mg L^−1^.

#### 2.6.4. Determination of Antioxidant Capacity

The AC of PCC extracts was evaluated using DPPH, ABTS, and FRAP assays according to procedures described by Cerda-Cejudo et al. [16].

For the DPPH assay, 7 μL of sample was mixed with 193 μL of reagent (2,2-diphenyl-1-picrylhydrazyl (DPPH, Sigma-Aldrich^®^, St. Louis, MO, USA) at 60 μM in absolute ethanol). The mixture was allowed to stand for 30 min, and the absorbance was measured at 517 nm using a microplate reader. Results were expressed as milligrams of Trolox equivalents per gram of dry matter (mg TE g dm^−1^), based on a standard Trolox curve from 0 to 200 mg L^−1^.

The ABTS reagent (2,2′-azinobis (3-ethylbenzothiazoline-6-sulfonate)) (Sigma-Aldrich^®^, St. Louis, MO, USA) was prepared by mixing 2.45 mL of 7 mM ABTS in absolute ethanol with 12.5 mL of 2.45 mM potassium persulfate (K_2_S_2_O_8_), followed by incubation in the dark for 16 h. After that, the absorbance was adjusted to 0.7 ± 0.2 at 734 nm. For the assay, 10 μL of extract was mixed with 190 μL of ABTS+ reagent, allowed to react for 1 min, and the absorbance was measured using a microplate reader. Results were expressed as Trolox equivalents per gram of dry matter (mg TE g dm^−1^) using a standard Trolox curve from 0 to 200 mg L^−1^.

The FRAP (ferric reducing antioxidant power) reagent consisted of acetate buffer solution (0.3 M, pH 3.6), 2,4,6-tri(2-pyridyl)-s-triazine (TPTZ Sigma-Aldrich^®^, St. Louis, MO, USA) 10 mM in 40 mM HCl, and ferric chloride (20 mM) mixed in a 10:1:1 ratio, and incubated at 37 °C for 30 min. For the assay, 18 μL of water, 6 μL of sample, and 180 μL of FRAP reagent were combined and incubated at 37 °C for 1 h before measuring absorbance at 595 nm using a microplate reader. Results were expressed as milligrams of Fe^+2^ per gram of dry matter (mg Fe^+2^ g dm^−1^) using a standard iron sulfate curve ranging from 0 to 800 mg L^−1^.

#### 2.6.5. Determination of Enzymatic Activity

*Cellulase (endoglucanase) activity:* Cellulase activity was determined according to the method described by Ascacio-Valdés et al. [17] with modifications. The assay consisted of mixing 200 µL of 200 ppm carboxymethylcellulose (substrate) with 50 µL of enzyme extract and incubating the mixture at 50 °C for 10 min. The enzyme blank contained 200 µL of substrate and 50 µL of 0.05 M citrate buffer (pH 5.0), whereas the substrate blank contained 200 µL of 0.05 M citrate buffer (pH 5.0) and 50 µL of enzyme extract. After incubation, the reaction was stopped by boiling for 5 min, followed by cooling in an ice bath for 1 min. Subsequently, 100 µL of the sample was mixed with 100 µL of DNS reagent (3,5-dinitrosalicylic acid), heated for 5 min, cooled, and diluted with 1 mL of distilled water. Finally, 200 µL of the mixture was transferred to a microplate, and reducing sugar was quantified at 540 nm using a dextrose standard curve (0–200 ppm). One unit of cellulase activity (U) was expressed as the amount of enzyme required to release 1 µmol of glucose per minute per gram of dry matter (U g dm^−1^).

*Xylanase activity:* For this assay, 200 µL of 500 ppm xylan substrate and 50 µL of enzyme extract were incubated at 50 °C for 10 min. The enzyme blank consisted of 200 µL of 0.05 citrate buffer (pH 5.0) and 50 µL of extract, whereas the substrate blank contained 200 µL of substrate and 50 µL of 0.05 citrate buffer (pH 5.0). After incubation, the reaction was stopped by boiling for 5 min, followed by cooling in an ice bath for 1 min. Afterwards, 100 µL of the sample was mixed with 100 µL of DNS reagent (3,5-dinitrosalicylic acid), boiled for 5 min, cooled, and diluted with 1 mL of distilled water. Then, 200 µL of the solution was transferred to microplate wells, and reducing sugars were measured at 540 nm using a microplate reader. One unit of xylanase activity (U) was defined as the amount of enzyme required to release 1 µmol of xylose per minute per gram of dry matter (U g dm^−1^) [9].

*β-glucosidase activity:* According to Izabal-Carvajal et al. [9] with modifications, the assay consisted of mixing 100 µL of 9 mM p-NPG (4-nitrophenyl β-D-glucopyranoside), 100 µL of enzyme extract and 800 µL of 0.05 mM citrate buffer (pH 5.0). The substrate blank contained 900 µL citrate buffer and 100 µL substrate, whereas the enzyme blank contained 100 µL enzyme extract and 900 µL citrate buffer. After boiling for 10 min, 100 µL of 0.1 M Na_2_CO_3_ was added. Subsequently, 100 µL of the sample was mixed with 100 µL of DNS reagent, boiled for 5 min, cooled in an ice bath for 1 min, and diluted with 1 mL of distilled water. From this solution, 200 µL was transferred to a microplate, and reducing sugars were measured at 400 nm using a microplate reader. One unit of β-glucosidase activity (U) was defined as the amount of enzyme to release 1 µmol of glucose per minute per gram of dry matter (U g dm^−1^).

*Tannase activity:* The assay consisted of mixing 250 µL of substrate (0.01 M methyl gallate) with 250 µL of enzyme extract. The substrate blank contained 250 µL of 0.05 mM citrate buffer (pH 5.0) and 250 µL of extract, whereas the enzyme blank contained 250 µL of substrate and 250 µL of 0.05 mM citrate buffer (pH 5.0). The mixture was incubated at 30 °C for 10 min, followed by the addition of 300 µL of methanolic rhodanine (0.667% *w*/*v*) and 200 µL of KOH (0.5 N). After adding 4 mL of distilled water, 200 µL of the sample was transferred to a microplate, and absorbance was measured at 520 nm. One unit of tannase activity (U) was defined as the amount of enzyme required to release 1 µmol of gallic acid per minute per gram of dry matter (U g dm^−1^) [9].

*Polyphenol oxidase (PPO) activity:* The methodology proposed by Izabal-Carvajal et al. [9] was followed, mixing 500 µL of 0.1 M pyrocatechol substrate with 500 µL of enzyme extract in test tubes. The substrate blank contained 500 µL of 0.05 mM citrate buffer (pH 5.0) and 500 µL of extract, whereas the enzyme blank contained 500 µL of substrate and 500 µL of 0.05 mM citrate buffer (pH 5.0). After adding 1.7 mL citrate buffer to all tubes, the mixtures were incubated at 30 °C for 10 min, boiled for 2 min, and cooled in an ice bath for 1 min. Finally, 200 µL of each sample was transferred to a microplate, and absorbance was measured at 420 nm using a microplate reader. One unit of polyphenol oxidase activity (U) was defined as the amount of enzyme required to produce 1 µmol of oxidized product (o-quinone or dopachrome) per minute per gram of dry matter (U g dm^−1^).

### 2.7. Identification of Phenolic Compounds by RP-HPLC-ESI-MS

An analysis was conducted using reversed-phase high-performance liquid chromatography, following the method of Cerda-Cejudo et al. [16], with modifications. A Varian HPLC system equipped with an automatic sampler (Varian ProStar 410, Palo Alto, Santa Clara, CA, USA), a ternary pump (Varian ProStar 230I, Palo Alto, CA, USA), and a diode array detector (Varian ProStar 330, Palo Alto, CA, USA), coupled to an ion trap mass spectrometer (Varian 500-MS IT, Palo Alto, CA, USA) equipped with an electrospray ionization source (ESI-MS). Samples (5 µL) were injected into a Denali C18 column (150 mm × 2.1 mm, 3 µm, Grace, Palo Alto, CA, USA) at 30 °C. The mobile phase consisted of formic acid (0.2%, *v*/*v*, solvent A) and acetonitrile (solvent B). The gradient used was: 3% B and 97% A; 0–5 min, 9% B and 91% A; 5–15 min, 16% B and 84% A; 15–45 min, 50% B and 50% A. The flow rate was 1.2 mL/min. Readings were recorded at 245, 280, 320, and 550 nm. Full-scan MS analysis was performed in negative mode [M-H]^−1^ over the *m*/*z* 50–2000 range, using nitrogen as the nebulizer gas and helium as the buffer gas. The capillary voltage was 90 V. Data were processed using MS Workstation software (V 6.9).

### 2.8. Statistical Analysis

A completely randomized 7 × 3 factorial design was used to evaluate the effects of fermentation time (0, 12, 24, 36, 48, 60, and 72 h) and forced aeration rate (0 and 1 L/Kg_wn_ min) on HP, CP, and AA of PCC. All experiments were performed in triplicate, and results were expressed as mean ± standard deviation. The coefficient of determination (R^2^) was used to assess the goodness of fit between the experimental CO_2_ production curves and those estimated by the model. Data were analyzed by analysis of variance (ANOVA) followed by Tukey’s test to determine significant differences among treatments. Pearson’s correlation coefficient was used to evaluate correlations among response variables at a significance level of *p* < 0.05. Statistical analyses were performed using SAS 9.0 software.

## 3. Results and Discussion

### 3.1. Physicochemical Characterization of PCC

The results of the proximate analysis of the PCC are presented in Table 1, revealing a composition of considerable biotechnological interest and highlighting its potential application as a fermentation substrate. The values obtained for fat, ash, proteins and moisture content were consistent with those reported by Guillen Sanchez and Siche [18] for PCC, with variations that may be attributed to differences in the corn variety from which the cob was derived.

The high carbohydrate content of PCC (67.2%) makes it an important carbon source for microbial growth, as carbohydrates serve as the primary energy source for microorganisms [19]. In addition, the elevated fiber content (21.37%) may promote greater cell density because the porous structure of corn cob fiber can function as a physical support for yeast cell immobilization. Furthermore, when combined with pretreatment that removes lignin, the substrate may facilitate the release of fermentable sugars required for the fermentation process [20]. Another relevant parameter is the fat content. Although PCC contains a relatively low fat concentration (0.38%) compared to other raw materials, lipids, together with fiber, can still provide an additional energy source for microorganisms. Moreover, lipids are essential components of the cell membrane, contributing to membrane functionality, permeability, cellular transport, and signaling processes [21]. The ash content (2.28%) reflects the presence of minerals in PCC, including phosphorus, magnesium, potassium, iron, and calcium. These minerals not only contribute to cellular balance and osmotic regulation, but also act as cofactors for enzymes involved in key metabolic pathways [22]. Although low, the protein content (2.51%) is the main source of nitrogen for microbial metabolism, and it is essential for the biosynthesis of macromolecules important for microbial growth, such as enzymes, amino acids, and nucleic acids [23].

PCC also exhibits a low moisture content (6.26%), which contributes to an extended shelf life and minimizes the risk of microbial contamination. However, in SSF, moisture content must generally be increased to 50–70%, depending on the requirements of the selected microbial strain) to facilitate nutrient transport and provide a suitable dissolution medium. These conditions are necessary to maintain enzymatic activity, metabolite mobility, and effective microbial colonization of the substrate while preventing osmotic stress [24,25].

Determination of hydrological parameters, such as WAC, CHP, and MMC, is essential for the design and optimization of SSF processes. These parameters help establish appropriate conditions that ensure microbial viability and activity while preserving substrate structure and aeration [26,27]. Table 2 presents the hydrological parameters of PCC evaluated to assess its suitability as a substrate for SSF.

According to Kanojia and Singh [28], WAC refers to the ability of plant materials or agro-industrial residues to absorb water relative to their own weight. This property depends on the gel-forming capacity of macromolecules and the availability of hydrophilic groups capable of binding water molecules. WAC is considered an important parameter in SSF because it reflects the ability of lignocellulosic materials to absorb and retain water, which directly influences microbial growth, nutrient diffusion, and metabolic activity under low free water conditions [29,30]. WAC values ranging from 2.97 to 12.09 g gel/g dm are considered favorable for promoting microbial growth in solid-state substrates [27]. The WAC value obtained for PCC (4.41 g gel/g dm) falls within the range reported by Buenrostro-Figueroa et al. [27] and Pérez-Díaz et al. [31] for white corn cobs (2.97 and 6.57 g gel/g dm, respectively). Similarly, this value is consistent with those reported for other agro-industrial residues successfully used in SSF processes, including rambutan peel (5.44), mango seed (3.4), pomegranate peel (4.38), pineapple peel (5.02), and pistachio shell (5.73) [12,15,32,33]. However, excessive high WAC values may increase the risk of substrate agglomeration, which can restrict airflow and oxygen diffusion, thereby limiting yeast growth and development [34].

The MMC represents the highest amount of moisture a material can retain under specific conditions without generating excess free water [16]. Excessive moisture levels decrease substrate porosity, reduce oxygen diffusion, increase the risk of contamination, and impair gas exchange. In contrast, low moisture levels can reduce nutrient solubility, limit substrate swelling, and increase water tension [25]. For PCC, an MMC value of 77.71% was obtained, which falls within the optimal moisture range (60–80%) reported for SSF processes [35].

The CHP indicates the percentage of water bound to the substrate, that is, water that is not available to microorganisms for metabolic activities. Therefore, lower CHP values are desirable to support microbial growth [36]. PCC exhibited a CHP value of 5.93 ± 0.85, which is lower than the values reported for white corn cobs (27%) and other substrates commonly used in SSF processes, such as mango seeds (46.2%), sugarcane bagasse (12%), coconut husk (16%), and candelilla stems (29.5%) [15,27]. This result suggests that PCC contains a lower proportion of bound water and, therefore, a greater amount of water available for microbial growth during SSF. Based on these findings, PCC exhibits physicochemical characteristics that support its suitability as a substrate for SSF processes.

### 3.2. Microbial Growth

*S. cerevisiae* 227 was used due to its established use in SSF studies and its demonstrated ability to enhance enzymatic activity and facilitate the release of bound phenolic compounds from plant-based substrates [9,10,33,37].

**Figure 2:**
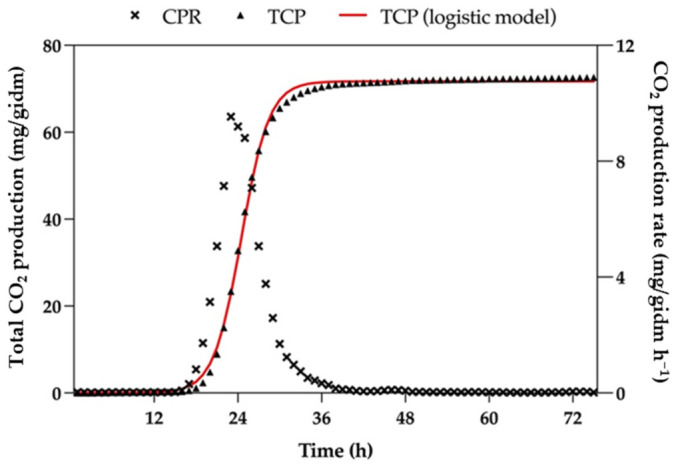
Respirometry profile of *S. cerevisiae* during SSF in PCC. CPR = CO_2_ production rate; TCP = total CO_2_ production; gidm = grams of initial dry mass.

The evolution of the SSF process was monitored through respirometric analysis. *S. cerevisiae* was able to grow using PCC as a substrate, reaching the maximum CO_2_ production rate (CPR) of 9.54 mg/gidm h^−1^ at 23 h. At the end of the SSF process, the total CO_2_ production (TCP) achieved by *S. cerevisiae* reached 71.70 mg/gidm^−1^. The TCP curves were described using Equation (6) (Figure 2), showing a high goodness of fit (R^2^ > 0.99) (Table 3). Respirometric analysis was performed only in the forced aerated system because continuous airflow was necessary to transport the reactor atmosphere to the CO_2_ detection system. In the non-forced aerated system, this analysis could not be conducted due to the absence of airflow required for gas transport to the detector.

Oxygen (O_2_) plays a critical role in the growth of *S. cerevisiae*, as it is required for the synthesis of sterols and unsaturated fatty acids, which are essential components of the cell membrane. Oxygen limitation inhibits growth and shortens the exponential phase; however, a controlled supply, such as continuous aeration, can prolong the growth phase and maintain cell viability. Nevertheless, oxygen delivery must be carefully regulated to prevent a metabolic shift toward excessive respiration, excessive biomass formation, or substrate bed drying caused by over-ventilation [38].

In SSF systems, mass and heat transfer are strongly influenced by substrate porosity and forced aeration, both of which facilitate CO_2_ removal and dissipation of metabolic heat. Inadequate aeration design may increase temperature gradients and promote moisture loss, leading to water stress and a shortened microbial growth phase. Conversely, the use of humidified air and continuous airflow can help maintain substrate water activity and bed stability, thereby extending the microbial growth period [39].

Maximum growth of *S. cerevisiae* was observed at 23 h of fermentation, coinciding with the highest CO_2_ production rate (CPR). Table 3 presents the respirometric parameters obtained from the evaluated fermentation kinetics.

The specific growth rate obtained using the logistic model (0.5039 h^−1^) was higher than the values reported by Castillo Plata [40] during the growth of *S. cerevisiae* and *Scheffersomyces stipitis* on hydrolyzed corn residues (0.137 h^−1^), as well as the value reported by Souza et al. [41] for *S. cerevisiae* fermenting corn substrates (0.2910 h^−1^). These differences may be primarily associated with variations in substrate composition. Although both studies used corn-derived materials, the exclusive use of corn cob in the present study may have provided conditions more favorable for yeast growth requirements [42]. Additionally, these variations may result from differences in the metabolic pathways activated during fermentation, leading to the production of secondary metabolites such as enzymes [32].

Similarly, Roukas [43] demonstrated that *S. cerevisiae* grown on carob pulp under SSF conditions exhibited optimal growth and yield at 70% moisture, 0.5 mm particle size, and 30 °C. Although aeration gradients were not evaluated in that study, the findings provide a useful reference for growth–time analysis and for assessing the potential effects of introducing aeration in similar fermentation systems (e.g., beds or trays). The authors reported that maximum biomass production occurred between 20 and 30 h of fermentation, which is consistent with the results obtained in the present study.

The early onset of the death phase observed in this study may be attributed to rapid nutrient depletion or to the formation of reactive oxygen species (ROS) during respiratory metabolism [44]. After approximately 24 h of carbon deprivation, yeast cells may lose a significant portion of their fermentative capacity due to ATP depletion. In addition, ROS generated during aerobic metabolism can damage essential cellular structures, including mitochondria, resulting in oxidative stress, reduced viability, and programmed cell death [45].

### 3.3. Effect of Fermentation on the Release of BPC

**Figure 3:**
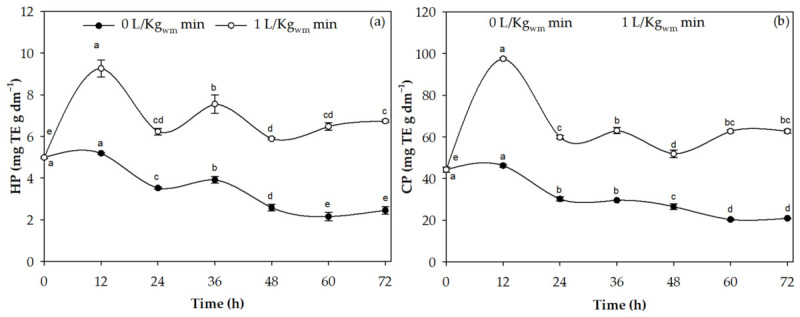
Effect of cultivation time and forced aeration on (**a**) Hydrolysable phenols (HP) and (**b**) Condensed phenols (CP) content in fermented PCC extracts. g dm^−1^ = gram of dry matter, mg GAE = milligrams of gallic acid equivalent, mg CE = milligrams of catechin equivalent. L/Kg_wm_ min = liters of air per kilogram of wet mass per minute. Different letters along the time indicate statistically significant differences.

The HP and CP contents, as a function of fermentation time and forced aeration level, are presented in Figure 3. The highest HP and CP values were observed at 12 h, showing increases of up to 1.76- and 2.03-fold, respectively, compared to the unfermented control (0 h). In addition, forced aeration exerted a positive effect, resulting in greater recovery of HP and CP, with increases of 1.78- and 2.1-fold, respectively. It is important to note that CP represented 91.32% of the TP content, indicating that this was the predominant phenolic fraction in PCC and the fraction exhibiting the greatest increase after SSF.

Following the peak release at 12 h, a decrease in phenolic content was observed at 24 h. This reduction may be attributed to the biotransformation of BPC, which can be modified or utilized as substrates for microbial development [12,15]. During the remaining fermentation period, phenolic values remained relatively stable. To date, no studies have reported the use of *S. cerevisiae* in SSF processes involving corn cobs. However, compared with studies using filamentous fungi, the results obtained in the present study are promising. Frumuzachi et al. [46] fermented corn husks using *A. niger* ATCC 6275 and reported a 2-fold increase in TP content. Similarly, SSF of corn grain with *Rhizopus oryzae* resulted in a 0.83-fold increase in BPC content [47].

Based on these findings, *S. cerevisiae* can be considered a microorganism capable of releasing polyphenolic compounds from plant matrices, specifically from PCC. This effect is attributed to the activity of cellulolytic and lignocellulolytic enzymes produced during SSF, which is consistent with the substrate composition [48]. These enzymes degrade structural components of the cell wall, thereby facilitating the release of bound phenolic compounds during microbial growth [16,32,49]. This phenomenon occurs during the early growth phase, generally between 2 and 32 h, when cell density increases. During this period, the presence of oxygen promotes the biosynthesis of essential cellular components, such as ergosterol and unsaturated fatty acids, which are critical for membrane structure and functionality. Consequently, yeast cells exhibit enhanced tolerance to ethanol and other metabolic stresses. Although *S. cerevisiae* can ferment under anaerobic conditions, an initial aerobic phase promotes greater biomass formation, resulting in higher cell density and improved fermentation performance in subsequent stages [50].

**Figure 4:**
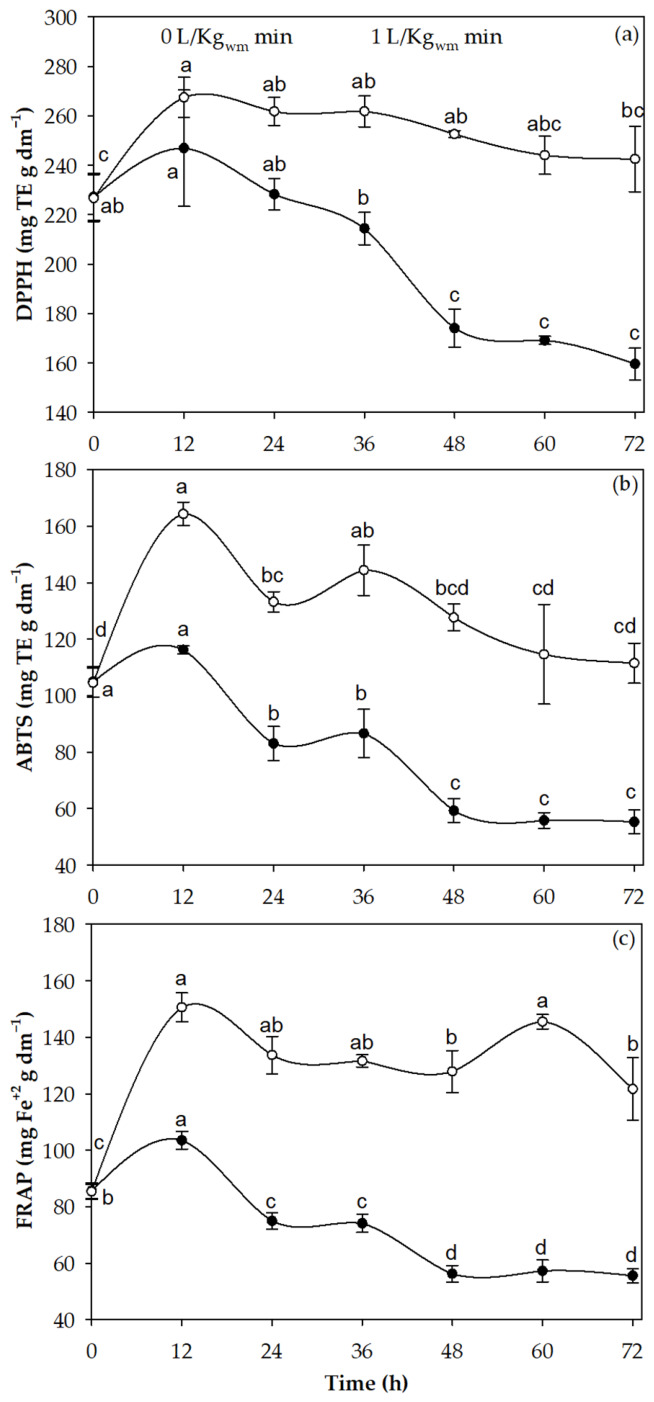
Effect of culture time and aeration on the antioxidant capacity of fermented extracts, using the DPPH (**a**), ABTS (**b**), and FRAP (**c**) assays. g dm^−1^ = gram of dry matter, mg TE= milligrams of Trolox equivalent, mgFe^+2^ = milligrams of ferrous ion. Different letters along the time indicate statistically significant differences.

The antioxidant capacity (AC) results of the fermented PCC extracts are shown in Figure 4. Similar to the BPC content, the highest AC values were observed at 12 h under forced aerated treatment, reaching 267.43 ± 8.11 mg TE g dm^−1^, 164.31 ± 4.12 mg TE g dm^−1^, and 150.59 ± 5.08 mg Fe^+2^ g dm^−1^ for the DPPH, ABTS, and FRAP assays, respectively. These values represent increases of up to 0.74-, 0.94-, and 0.64-fold, respectively, compared to the unfermented control. These increases were greater than those reported for fermented corn grain, which showed increases of 1.04-, 1.1-, and 1.76-fold for the DPPH, ABTS, and FRAP assays, respectively [47]. This behavior further supports the association between the BPC and its AC, suggesting that the increase in AC is directly related to the higher BPC levels released during the SSF process.

### 3.4. Effect of SSF on Enzyme Production

**Figure 5:**
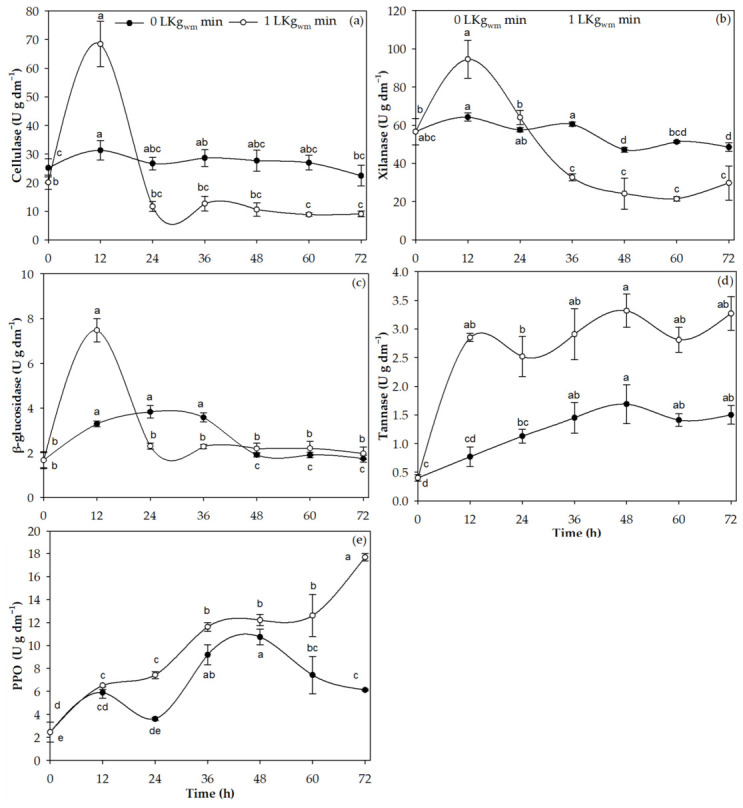
Effect of aeration and cultivation time on the production of (**a**) cellulase, (**b**) xylanase, (**c**) *β*-glucosidase, (**d**) tannase, and (**e**) polyphenol oxidase from PCC fermented with *S. cerevisiae.* PPO = Polyphenol oxidase, U g dm^−1^ = Units per gram of dry matter. Different letters along the time indicate statistically significant differences.

During the SSF process of PCC with *S. cerevisiae*, the activity of several enzymes of industrial interest was evaluated (Figure 5), as these enzymes have been associated with the biodegradation of BPC [9,17,51]. The highest cellulase activity was detected at 12 h of fermentation (Figure 4a). Notably, forced aeration significantly enhanced enzyme production, reaching 68.48 ± 7.8 U g dm^−1^, which represented a 3.4-fold increase compared to the non-forced aerated treatment (0 L/Kg_wm_ min). This result was consistent with both the maximum release of BPC and the highest AC values observed during fermentation. A marked decrease in cellulase activity (11.71 ± 1.75 U g dm^−1^) was observed at 24 h. This reduction may be attributed to several factors, including product inhibition by cellobiose and catabolic repression associated with the Crabtree effect. In the presence of excess glucose, glycolytic activity increases, and yeast cells obtain energy primarily through fermentation rather than respiration, thereby reducing the metabolic resources for enzyme synthesis [52].

Additionally, enzymatic degradation may also contribute to the decline in cellulase activity due to the action of proteases produced during the stationary or stress phase. When glucose or essential nutrients become limited, *S. cerevisiae* activates autophagy and proteolysis pathways to recycle intracellular proteins and maintain cell viability [53]. Amadi et al. [54] reported a cellulase activity of 41 U g dm^−1^ at 144 h during the SSF of corn cob using *S. cerevisiae* SCPW 17, which was a value 1.31-fold lower than the one obtained in the present study and achieved after a cultivation period 12 times longer. According to Mahalakshmi and Jayalakshmi [55], maximum cellulase production generally occurs during the exponential phase. Therefore, the low cellulase activity observed under non-forced aeration conditions was not unexpected, particularly considering the limited evidence regarding the intrinsic capacity of yeasts to produce cellulolytic enzymes [56].

The behavior of xylanase activity during SSF is presented in Figure 5b. The highest activity was observed at 12 h under forced aerated conditions, reaching 94.57 ± 9.93 U g dm^−1^. This value was 1.47-fold higher than that obtained under non-forced aeration conditions and 1.66-fold higher than the non-fermented control. Between 24 and 36 h of fermentation, xylanase activity decreased to 32.71 ± 1.79 U g dm^−1^. This reduction may be attributed to the mechanisms previously discussed, including product inhibition, catabolic repression, and protease activity. Protease production is strongly influenced by physicochemical and nutritional factors, including aeration [57]. Therefore, its presence in a forced aeration system may contribute to the degradation or inactivation of other enzymes, such as cellulases and xylanases. In the non-forced aeration conditions, the production of both cellulases and xylanases was minimal, and their activities tended to decline over time.

The xylanase activity obtained in this study exceeded that reported by Amadi et al. [54], who documented 52 U g dm^−1^ at 144 h during submerged fermentation of corn cob with *S. cerevisiae* SCPW 17. This difference may be associated with the effect of forced aeration. Oxygen availability is essential for xylose metabolism in yeasts because it activates mitochondrial respiration [58], resulting in a substantially higher ATP yield per mole of sugar compared to fermentative metabolism (36–38 vs. 2 ATP). Furthermore, in substrates rich in fermentable sugars, oxygen reduces ethanol production and redirects metabolism toward respiration, thereby promoting biomass accumulation and enzyme synthesis [59].

Similar to cellulases, xylanases are not constitutively produced enzymes. Therefore, their synthesis is influenced primarily by substrate composition rather than representing an intrinsic characteristic of yeast metabolism. Given that corn cobs contain high levels of hemicellulose (20–25%), they may act as inducers of xylanolytic activity [54]. Two main factors may explain the persistence of these enzymes. First, continuous substrate induction: the high xylan content stimulates xylanolytic enzyme expression, as previously observed in *A. niger*, where maintaining adequate substrate levels supports xylanase production over extended periods [60]. In *S. cerevisiae*, corn cobs likely function both as a structural matrix and as an inducer, facilitating enzyme release and relatively stable enzyme production [54]. Second, under oxygen-limited conditions, yeast metabolism may activate alternative gene expression pathways that enable the utilization of hemicellulosic components as secondary carbon sources. Additionally, the conversion of xylose to xylitol may alleviate inhibitory effects, thereby buffering pH and allowing continued xylanase secretion [61].

The production profile of β-glucosidase (Figure 5c) showed maximum activity at 12 h under forced aerated conditions, reaching 7.48 ± 0.51 U g dm^−1^. This value represented a 4.47-fold increase compared to the unfermented control and a 1.95-fold increase relative to the non-forced aerated conditions at the same cultivation time. As observed for cellulase and xylanase activities, β-glucosidase activity declined at 24 h. This reduction may be attributed to yeast metabolic regulation and to inhibitory compounds present in PCC, such as lignin and ferulic acid, which can limit substrate accessibility and interfere with cellular energy metabolism [62,63,64]. During the SSF of pomegranate peel with *S. cerevisiae*, Izábal-Carvajal et al. [9] reported β-glucosidase activity of 2.45 U g dm^−1^ after 36 h of cultivation. In comparison, the values obtained in the present study were 1.56-fold higher under non-forced aerated conditions and 3.05-fold higher under forced aerated conditions. These findings are consistent with Tang et al. [65], who demonstrated that β-glucosidase production by *S. cerevisiae* is enhanced in the presence of oxygen.

Tannase activity (Figure 5d) further confirmed the positive effect of aeration on enzyme production. Throughout the SSF process, higher tannase levels were consistently observed in the forced aerated conditions than in the non-forced aerated treatment. Under forced aeration, maximum activity was maintained between 12 and 72 h, with no statistically significant differences among these time points (*p* ≤ 0.05). Nevertheless, the highest numerical value was recorded at 48 h (3.32 ± 0.28 U g dm^−1^), corresponding to an 8.3-fold increase compared to the unfermented control.

In contrast, the non-forced aerated treatment exhibited a more gradual and sustained production pattern. Although overall activity was lower, the maximum value was observed at 48 h, representing a 4.22-fold increase relative to the control, but 1.92-fold lower than the value obtained under forced aerated conditions at the same time point.

Aharwar and Parihar [66] reported that tannase production has been primarily associated with filamentous fungi, whereas only a limited number of yeast strains (such as *Pichia* sp. and *S. cerevisiae*) have demonstrated this capability. Indeed, reports describing tannase production by *S. cerevisiae* are scarce compared to those involving filamentous fungi [67]. However, the present findings indicate that the *S. cerevisiae* 227 strain is capable of producing tannase and that enzyme productivity may be further enhanced through process optimization strategies.

In the non-forced aerated treatment, the highest PPO activity (Figure 5e) was observed between 36 and 48 h, reaching 10.74 ± 0.69 U g dm^−1^, which represented a 4.37-fold increase compared to the unfermented control. In contrast, under forced aerated conditions, PPO activity increased progressively throughout cultivation, with notable production peaks at 12 and 24 h and a maximum value at 72 h (17.67 ± 0.32 U g dm^−1^), corresponding to a 7.18-fold increase relative to the control. Overall, forced aeration enhanced PPO production by approximately 64% compared to the non-forced aerated treatment. This effect may be attributed to improved oxygen availability within the solid matrix. Although *S. cerevisiae* is a facultative microorganism, PPO is an oxidative enzyme that requires molecular oxygen to catalyze the oxidation of bound phenolic compounds (BPC) [68]. Therefore, oxygen availability directly supports enzymatic activity and the associated phenolic transformations, including the hydroxylation of monophenols to diphenols and the oxidation of catechol to quinones [69]. In addition, oxygen sustains metabolic pathways involved in phenolic degradation and promotes efficient enzyme expression [70]. Conversely, oxygen limitation restricts metabolic activity and reduces enzyme synthesis. Beyond its biochemical role, forced aeration improves process control by dissipating metabolic heat, thereby minimizing the risk of enzyme denaturation [71]. In addition. forced aeration facilitates humidity regulation and CO_2_ removal, preventing bed compaction and the development of anaerobic microenvironments that could impair enzyme activity. The removal of accumulated CO_2_ further enhances yeast respiratory metabolism, thereby increasing enzymatic productivity [68,72,73].

It is important to highlight that SSF represents a valuable platform for the production of enzymes with broad industrial applications, offering a sustainable alternative to conventional chemical processes that are often more costly and environmentally detrimental. For example, cellulases are widely used in the paper, detergent, food, and biofuel industries [74], whereas xylanases have applications in food processing, textiles, and biofuel production [75]. PPO, although not directly exploited at large-scale industrial production, is relevant in studies focused on enzymatic browning and its control in foods [76]. Tannases are commonly used in the clarification and stabilization of wines, juices, and teas, as well as in the production of gallotannins with nutraceutical and pharmaceutical applications [77]. β-glucosidases are essential in winemaking for the release of aromatic compounds and are also employed in fermented beverage and bakery processes [63].

Overall, SSF emerges as a promising biotechnological strategy for producing high-value enzymes from agro-industrial residues [73], including PCC. This approach not only promotes by-product valorization but also contributes to replacing environmentally detrimental chemical processes, thereby aligning with the principles of circular economy and sustainable bioprocessing [78].

### 3.5. Correlation Between BPC Content, AC and Enzymatic Activities

**Figure 6:**
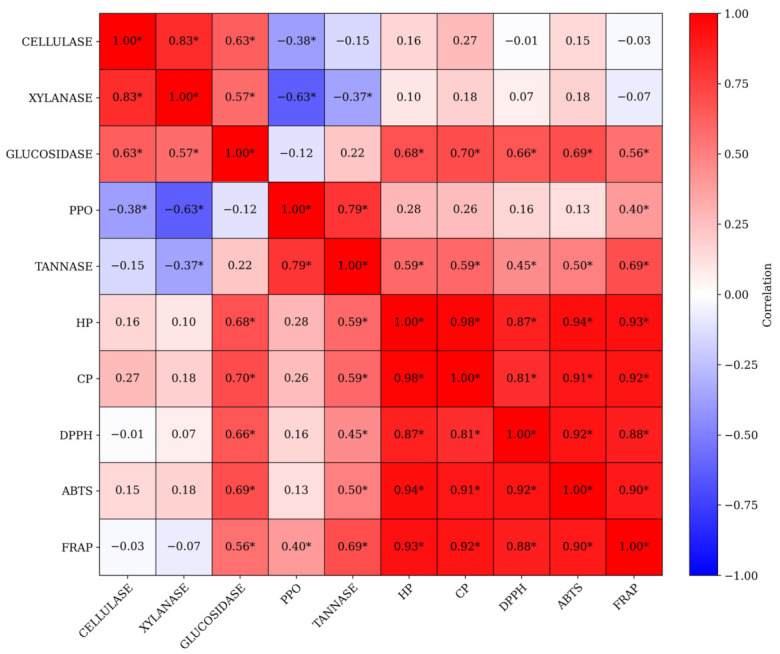
Pearson correlation matrix among HP, CP, antioxidant capacity determined by DPPH, ABTS and FRAP assays, and enzymatic activities. The color gradient indicates the magnitude and direction of the correlations (red: positive, blue: negative), while asterisks (*) denote statistically significant associations. PPO = Polyphenol oxidase, HP = hydrolysable phenols, CP = condensed phenols.

Pearson’s correlation coefficient (*r*) was used to statistically evaluate the relationships among BPC, AC, and the enzymatic activities detected in PCC extracts fermented with *S. cerevisiae*. The results are presented in Figure 6. A very strong positive correlation was observed between HP and AC measured by DPPH, ABTS, and FRAP assays, with *r* values of 0.87, 0.94, and 0.93, respectively. These results indicate that increases in HP content are closely associated with enhanced antioxidant potential of the extracts.

Similarly, when considering the predominant phenolic fraction (CP), strong positive linear correlations were observed with DPPH (*r* = 0.81), ABTS (*r* = 0.91), and FRAP (*r* = 0.92). These findings suggest that CP plays a major role in determining the antioxidant performance of fermented extracts and that increases in CP concentration directly contribute to greater radical-scavenging and reducing capacities. In contrast, correlations between enzymatic activities and BPC content varied among enzymes, indicating that each enzymatic system may contribute differently to phenolic release and transformation during SSF. These differential associations highlight the complexity of the enzymatic mechanisms involved in lignocellulosic degradation and phenolic compound liberation.

Cellulase activity showed a weak, non-significant positive correlation with BPC release and, consequently, with AC. Mechanistically, cellulase catalyzes the hydrolysis of β-1,4-glycosidic linkages in cellulose and contributes to the disruption of hemicellulosic structures present in corn cobs. These structural degradations facilitate the release of BPC, including ferulic and *p*-coumaric acids [79]. However, the modest strength of the correlation suggests that cellulase alone may not be sufficient to drive substantial phenolic liberation; rather, it functions as part of a broader enzymatic system.

Xylanase is a hydrolase that cleaves β-1,4-linked D-xylopyranose units within the heteropolymeric structure of xylan [80]. This enzyme contributes to the overall deconstruction of the lignocellulosic matrix by hydrolyzing hemicellulose into xylooligosaccharides and monomeric sugars, while partially disrupting lignin–hemicellulose interactions [81]. The principal hydrolysis products are xylooligosaccharides and xylose, both of considerable biotechnological interest. Similar to cellulase, xylanase exhibited a very weak and non-significant correlation with BPC release and AC. This finding is consistent with the fact that xylanase does not directly generate phenolic compounds as reaction products [82]. Comparable results were reported by Wang et al. [83], who observed that xylanase alone did not significantly enhance BPC content or AC, as measured by DPPH, ABTS, and FRAP assays, in guava leaf extracts. However, when combined with cellulase and other lignocellulolytic enzymes, a synergistic effect was detected. This behavior suggests that the release of phenolic compounds bound to polysaccharides depends on coordinated enzymatic pathways and that incomplete hydrolysis of the lignocellulosic cell wall may restrict access to these compounds [84].

β-glucosidase and tannase were the enzymes most strongly associated with increases in BPC content and AC, showing significant positive correlations (*p* ≤ 0.05) ranging from 0.56 to 0.70 and 0.45 to 0.69, respectively. These results indicate that higher enzymatic activity is directly associated with greater phenolic release and antioxidant potential in PCC extracts. In substrates rich in phenolic glycosides, β-glucosidase plays an important role by hydrolyzing β-glycosidic bonds (β-1,4) present in flavonoids, phenylpropanoids, and other secondary metabolites, such as quercetin or resveratrol, potentially releasing bioactive aglycones with greater AC [85,86].

Previous studies have similarly reported that increased β-glucosidase activity enhances AC, as measured by DPPH, ABTS, and FRAP assays, attributing this effect to the liberation of bound aglycones [83]. However, the magnitude of the antioxidant response depends on compound polarity. Aglycone flavonoids, being more lipophilic, interact more efficiently with ABTS radicals, whereas glycosylated forms are more hydrophilic and may show different affinities in ABTS and FRAP assays [87,88]. Phenylpropanoids, depending on their substituents, may exhibit moderate hydrophobicity, favoring interaction with DPPH radicals [89]. Therefore, the observed correlations between β-glucosidase activity and AC are influenced not only by phenolic release but also by the physicochemical properties of the hydrolyzed compounds.

In the case of tannase, this enzyme acts as an esterase that degrades tannins, releasing gallic acid, catechin, and epigallocatechin [90]. These compounds are widely recognized for their high AC [91]. Accordingly, several studies have reported that SSF of agro-industrial by-products enhances both BPC content and AC, as complex tannins are biotransformed into smaller, more soluble, and more reactive phenolics, such as gallic acid [92]. This mechanism supports the significant positive correlations observed between tannase activity, BPC content, and AC in the present study.

Finally, PPO is an oxidoreductase that catalyzes the oxidation of monophenols to *o*-diphenols and subsequently to *o*-quinones in the presence of molecular oxygen [93]. In this study, PPO activity showed a weak and non-significant positive correlation with BPC release and overall AC (*r* = 0.13–0.28). However, a significant positive correlation was observed between PPO and AC measured by the FRAP assay (*r* = 0.40). Although PPO is commonly associated with enzymatic browning, it can also modify phenolic structures reported in PCC, such as anthocyanins and flavonoids [94]. These oxidative transformations may generate intermediate or derivative molecules with enhanced reducing capacity, thereby increasing FRAP values [95]. Thus, beyond its role in browning reactions, PPO may indirectly influence the antioxidant profile of fermented extracts through structural modification of phenolic substrates.

It should be noted that Pearson’s correlation coefficient evaluates only linear pairwise associations and does not account for causal relationships or the complex multivariate interactions characteristic of multienzymatic systems. Therefore, weak or non-significant correlations do not necessarily indicate biological irrelevance, but may instead reflect indirect, synergistic, or nonlinear interactions among variables.

### 3.6. Identification of Phenolic Compounds

A total of 33 BPC were identified in PCC extracts obtained during the SSF process (Table 4). Of these, 15 compounds were detected in the unfermented control at the initial sampling time, whereas the remaining compounds were generated or became detectable throughout the bioprocess. The phenolics compounds identified at the initial time included sesamolinol, caffeic acid 4-O-glucoside, rhamnetin, ferulic acid 4-*O*-glucoside, 3-feruloylquinic acid, luteolin 6-C-glucoside, 4-feruloylquinic acid, 5-feruloylquinic acid, petunidin 3,5-*O*-diglucoside, cyanidin 3,5-*O*-diglucoside, isorhamnetin 3-*O*-glucoside 7-*O*-rhamnoside, galoyl-HHDP-hexoside, 5,6-dihydroxy-7,8,3′,4′-tetramethoxyflavone, and *p*-coumaric acid 4-*O*-glucoside.

During SSF with *S. cerevisiae*, 18 additional BPC were detected, 72% of which appeared at 12 h of fermentation, corresponding to the time point at which the highest total BPC content, AC, and most enzymatic activities were recorded. This temporal coincidence supports the central role of enzymatic hydrolysis in the release and transformation of phenolic compounds during SSF. Compounds generated at this stage included (+)-catechin, 3,4-DHPEA-EA, 1-caffeoylquinic acid, scopoletin, delphinidin 3-*O*-glucoside, isorhamnetin 3-*O*-glucoside, delphinidin 3-*O*-feruloylglucoside, luteolin 7-*O*-rutinoside, 1-sinapoyl-2-feruloylgentiobiose, petunidin 3-*O*-glucoside, scottenol ferulate, 5-*O*-galoylquinic acid, and oleuropein aglycone.

As fermentation progressed, several compounds initially present in the control decreased or disappeared, whereas others emerged, suggesting oxidation, hydrolysis, or structural transformation into smaller phenolic derivatives. These changes are consistent with the enzymatic cleavage of ester, ether, and glycosidic bonds linking phenolics to the lignocellulosic matrix. Moreover, the solvent system used may influence compound detectability, since phenolics soluble in organic solvents are typically present in free or conjugated forms [6]. During SSF, enzymes such as esterases, laccases, xylanases, and cellulases, produced under the conditions evaluated in this study, contribute to the degradation of cellulose and hemicellulose, thereby exposing and releasing bound phenolic compounds [4,96,97].

Pigmented corn tissues are known to contain a complex phenolic profile dominated by anthocyanins (cyanidin, pelargonidin), peonidin-based glycosides and their malonylated derivatives, as well as phenolic acids (including caffeic acid derivatives and p-coumaric acid), flavonoids (such as apigenin and luteolin glycosides), and hydroxycoumarins such as scopoletin [94]. Comparable anthocyanin patterns have been documented in purple corn varieties, where cyanidin-3-glucoside is frequently identified as a major contributor to pigmentation and antioxidant potential [96].

Anthocyanins are widely used as natural food colorants due to their chromatic properties and AC, serving as functional alternatives to synthetic dyes [98]. Beyond their technological applications, these compounds exhibit important pharmacological activities, including antithrombotic, cardioprotective, and potential anticancer effects. For example, delphinidin-3-*O*-glucoside has demonstrated antithrombotic activity [99]. Similarly, flavonols possess well-documented antioxidant and anti-inflammatory properties, supporting their application in pharmaceutical, nutraceutical, and cosmetic formulations [100]. Some compounds within this group have also demonstrated significant antihypertensive activity, reinforcing their potential incorporation into functional food systems [101].

Rosmanol (compound 10) was consistently detected throughout the SSF process. Although this diterpenoid phenolic compound was previously reported by Ramírez-Esparza et al. [102] during SSF of blue corn using *Rhizopus oryzae*, it is not commonly considered as a natural constituent of corn matrices, but rather of plants such as rosemary [103]. In the presence study, compound annotation was based solely on *m*/*z* values and retention times obtained by HPLC-MS analysis, without confirmation using an authentic analytical standard. Therefore, its identification should be considered tentative, and further confirmation through analytical standards and/or complementary techniques such as Nuclear Magnetic Resonance is necessary to unequivocally confirm its identity.

The detection of caffeoylquinic acid and *p*-coumaric acid is particularly significant because these phenolic acids are commonly present in free or esterified forms within the plant cell wall matrix. Their release during fermentation likely reflects enzymatic hydrolysis of bound phenolic esters under conditions favoring partial degradation of the cell wall [104]. Caffeoylquinic acid is recognized as a multifunctional compound with preservative properties and potential use in edible coatings [105], as well as antioxidant, anti-inflammatory, antiviral, antidiabetic, anti-obesity, and hepatoprotective activities [106]. In biotechnology, it also has applications in nanoparticle synthesis, electrode modification, and the development of natural colorants [107]. Likewise, *p*-coumaric acid exhibits antioxidant and antimicrobial properties and is used as a natural preservative in functional foods [108], in addition to possessing anti-inflammatory, antiviral, and anticancer potential [109].

Three additional compounds (cyanidin 3,5-*O*-diglucoside, galloyl-HHDP-hexoside, and 5,6-dihydroxy-7,8,3′,4′-tetramethoxyflavone) persisted throughout the SSF process. These molecules share antioxidant, anti-inflammatory, and anticancer activities, highlighting their relevance for applications in the pharmaceutical, cosmetic, and food industries [110,111].

Overall, these results demonstrate that PCC represents a suitable substrate for the generation, release, and transformation of diverse phenolic compounds, as confirmed by HPLC analysis. The recovery of molecules with antioxidant, anti-inflammatory, and anticancer activities underscores their industrial relevance. Importantly, their production through SSF demonstrates the potential of *S. cerevisiae* to valorize agro-industrial residues such as PCC by promoting enzymatic release and structural modification of bioactive phenolics through an economically viable, environmentally sustainable bioprocess.

## 4. Conclusions

The physicochemical properties and hydrological parameters of PCC favor microbial colonization, highlighting its potential suitability for sustainable biotechnological applications. PCC proved to be an effective substrate for SSF, supporting the growth of *S. cerevisiae* and enabling both enzyme production and the release of BPC.

Fermentation resulted in a significant enhancement of phenolic release and AC, with increases of up to 216 and 94%, respectively, after 12 h of cultivation. This peak coincided with maximum enzymatic activity, particularly for xylanase and cellulase, suggesting that lignocellulosic matrix degradation played a central role in the liberation of bound phenolics. The detection of β-glucosidase and tannase further supports the occurrence of enzymatic hydrolysis and biotransformation processes during SSF.

HPLC–MS analysis confirmed qualitative changes in the phenolic profile, including the release and/or formation of compounds such as caffeic acid, caffeoylquinic acid, and p-coumaric acid, which are recognized for their antioxidant and multifunctional industrial applications. These findings indicate that SSF not only enhances phenolic availability but also modulates the metabolite composition of the substrate.

Overall, PCC can be revalorized through SSF as part of a microbial biorefinery strategy, enabling the conversion of agro-industrial residues into high-value bioactive compounds and industrial enzymes. This approach may contribute to the development of sustainable bioprocesses and aligns with circular economy principles by promoting waste valorization and environmentally responsible production systems.

## Figures and Tables

**Table 1:** Proximate analysis of the PCC (%, dry matter).

Parameter	Value (%)
Fat	0.38 ± 0.06
Ash	2.28 ± 0.12
Protein	2.51 ± 0.17
Moisture	6.26 ± 0.62
Fiber	21.37 ± 1.1
Carbohydrates	67.2 ± 0.58

The values indicate the mean ± standard deviation of 3 replicates.

**Table 2:** Hydrological characterization of PCC.

Parameters	Results
Water absorption capacity (WAC; g gel/g dm)	4.41 ± 0.08
Maximum moisture content (MMC; %)	77.71 ± 1.65
Critical humidity point (CHP; %)	5.93 ± 0.85

g gel/g dm: grams of gel per gram of dry matter.

**Table 3:** Respirometry parameters of *S. cerevisiae* in SSF of PCC.

Parameter (Logistic Model)	Value
µ (h^−1^)	0.5039
CO_2o_ (mg gidm^−1^)	0.0005
CO_2max_ (mg gidm^−1^)	71.7018
t_lag_	16.9 h
R^2^ _adj_	0.9992

µ = Specific CO_2_ production rate; CO_2o_ = Initial CO_2_ production; CO_2max_ = Total CO_2_ production.

**Table 4:** Identification of compounds via HPLC-MS during SSF of PCC with *S. cerevisiae*.

	Retention Time (min)	[M-H]^−^	Compound	Molecular Formula	Family	Fermentation Time (h)						
						0	12	24	36	48	60	72
1	3.809	370.9	Sesamolinol	C_20_H_20_O_7_	Lignans	x						
2	3.897	288.8	(+)-Catechin	C_15_H_14_O_6_	Catechins		x	x	x	x	x	x
3	5.021	376.8	3,4-DHPEA-EA	C_19_H_22_O_8_	Tyrosols		x	x	x	x	x	
4	5.47	352.8	1-caffeoylquinic acid	C_16_H_18_O_9_	Hydroxycinnamic acids		x	x	x		x	x
5	5.668	341.6	Caffeic acid 4-*O*-glucoside	C_15_H_18_O_9_	Hydroxycinnamic acids	x						
6	6.611	190.9	Scopoletin	C_10_H_8_O_4_	Hydroxycoumarins		x					
7	8.003	314.9	Rhamnetin	C_16_H_12_O_7_	Methoxyflavonols	x	x					
8	9.087	223.4	Sinapic acid	C_11_H_12_O_5_	Methoxycinnamic acids					x		
9	10.45	332	Gallic acid 4-*O*-glucoside	C_13_H_16_O_10_	Hydroxybenzoic acids				x			
10	18.275	344.9	Rosmanol	C_20_H_26_O_5_	Phenolic terpenes	x	x	x	x	x	x	x
11	21.041	464.8	Delphinidin 3-*O*-glucoside	C_21_H_21_O_12_	Anthocyanins		x					
12	21.542	354.8	Ferulic acid 4-*O*-glucoside	C_16_H_20_O_9_	Methoxycinnamic acids	x						
13	22.78	367.9	3-Feruloylquinic acid	C_17_H_20_O_9_	Methoxycinnamic acids	x		x	x	x	x	x
14	23.496	446.9	Luteolin 6-C-glucoside	C_21_H_20_O_11_	Flavones	x					x	
15	23.643	488.8	Quercetin 3-*O*-acetyl-rhamnoside	C_23_H_22_O_12_	Flavonols			x	x	x	x	x
16	23.981	478.8	Isorhamnetin 3-*O*-glucoside	C_22_H_22_O_12_	Methoxyflavonols		x				x	x
17	26.332	488.7	Kaempferol 3-*O*-acetyl-glucoside	C_23_H_22_O_12_	Flavonols			x				
18	26.378	366.8	4-Feruloylquinic acid	C_17_H_20_O_9_	Methoxycinnamic acids	x	x	x	x			
19	30.46	638.7	Delphinidin 3-*O*-feruloyl-glucoside	C_31_H_29_O_15_	Anthocyanins		x					
20	31.36	610	Cyanidin 3-*O*-sambubioside	C_26_H_29_ClO	Anthocyanins			x		x		
21	32.664	366.8	5-Feruloylquinic acid	C_17_H_20_O_9_	Methoxycinnamic acids	x	x					
22	34.096	638.9	Petunidin 3,5-*O*-diglucoside	C_28_H_33_ClO_17_	Anthocyanins	x	x	x				
23	35.332	592.7	Luteolin 7-*O*-rutinoside	C_27_H_30_O_15_	Flavones		x	x		x		
24	36.261	723.1	1-Sinapoyl-2-feruloylgentiobiose	C_22_H_23_O_12_Cl	Methoxycinnamic acids		x	x	x	x	x	x
25	37.155	476.8	Petunidin 3-*O*-glucoside	C_22_H_23_O_12_	Anthocyanins		x					
26	37.345	608.9	Cyanidin 3,5-*O*-diglucoside	C_27_H_30_O_16_	Anthocyanins	x	x	x	x	x	x	x
27	40.788	622.9	Isorhamnetin 3-*O*-glucoside 7-*O*-rhamnoside	C_28_H_32_O_16_	Methoxyflavonols	x	x	x	x	x		
28	41.425	588.6	Schottenol ferulate	C_39_H_58_O_4_	Methoxycinnamic acids		x	x	x		x	x
29	42.741	342.9	5-*O*-Galloylquinic acid	C_14_H_16_O_10_	Hydroxybenzoic acids		x	x			x	x
30	43.319	632.8	Galloyl-HHDP-hexoside	C_41_H_28_O_2_	Polyphenols	x	x	x	x	x	x	x
31	44.335	372.8	5,6-Dihydroxy-7,8,3′,4′-tetramethoxyflavone	C_19_H_18_O_8_	Methoxyflavones	x	x	x	x	x	x	x
32	45.803	376.8	Oleuropein-aglycone	C_19_H_22_O_8_	Tyrosols		x	x	x	x	x	x
33	48.436	325.0	p-Coumaric acid 4-*O*-glucoside	C_15_H_18_O_8_	Hydroxycinnamic acids	x	x	x	x	x	x	x

## Data Availability

The original contributions presented in this study are included in the article. Further inquiries can be directed to the corresponding authors.

## Abbreviations

The following abbreviations are used in this manuscript:

PCC	Pigmented corn cobs
BPC	Bioactive phenolic compounds
SSF	Solid-state fermentation
AC	Antioxidant capacity
HPLC-MS	High-performance liquid chromatography coupled with mass spectrometry
AOAC	Association of Official Analytical Chemists
WAC	Water absorption capacity
CHP	Critical humidity point
MMC	Maximum moisture content
g	Grams
mg	Milligrams
mL	Milliliters
min	Minutes
°C	Degrees Celsius
g gel/g dm	Grams of gel per gram of dry matter
PDA	Potato-dextrose agar
*v*/*v*	Volume/Volume
g/L	Grams per liter
NaCl	Sodium chloride
cm	Centimeters
h	Hour
L/Kg_wm_ min	Liters of air per kilogram of wet mass per minute
µm	Micrometers
CO_2_	Carbon dioxide
O_2_	Oxygen
rpm	Revolutions per minute
M	Molar
CPR	CO_2_ production rate
gidm	Grams of initial dry matter
TCP	Total CO_2_ production
Ln	Natural logarithm
CO_2o_	Initial CO_2_ production
CO_2max_	Total CO_2_ production
HP	Hydrolysable phenols
µL	Microliters
N	Normal
nm	Nanometers
GAE	Gallic acid equivalents
g dm^−1^	Gram of dry matter
mg L^−1^	Milligrams per liter
HCl	Hydrochloric acid
CE	Catechin equivalents
μM	Micromolar
TE	Trolox equivalents
mM	MilliMolar
K_2_S_2_O_8_	Potassium persulfate
DPPH	2,2-diphenyl-1-picrylhydrazyl
ABTS	2,2’-azinobis (3-ethylbenzothiazoline-6-sulfonate)
FRAP	Ferric reducing antioxidant power
TPTZ	2,4,6-tri(2-pyridyl)-s-triazine
ppm	Parts per million
DNS	3,5-dinitrosalicylic acid
p-NPG	4-nitrophenyl β-D-glucopyranoside
Na_2_CO_3_	Sodium carbonate
w	Weight
v	Volume
KOH	Potassium hydroxide
RP-HPLC-ESI-MS	Reverse-phase high-performance liquid chromatography–electrospray ionization Mass spectrometry
mm	Millimeters
m/z	Mass-to-charge ratio
R^2^	Coefficient of determination
ANOVA	Analysis of Variance
CPR	Maximum CO_2_ production rate
µ	Specific CO_2_ production rate
ROS	Reactive oxygen species
ATP	Adenosine triphosphate
CP	Condensed phenols
TP	Total phenols
Fe^+2^	Ferrous ion
PPO	Polyphenol oxidase
HHDP	Hexahydroxydiphenoyl
